# Effect of Silicon Carbide and Tungsten Carbide on Concrete Composite

**DOI:** 10.3390/ma15062061

**Published:** 2022-03-10

**Authors:** Maria Idrees, Husnain Ahmad Chaudhary, Arslan Akbar, Abdeliazim Mustafa Mohamed, Dina Fathi

**Affiliations:** 1 Department of Architectural Engineering & Design, University of Engineering &Technology, Lahore 54000, Pakistan; mariaidrees@uet.edu.pk (M.I.); husnainking1092@gmail.com (H.A.C.); 2 Department of Architecture and Civil Engineering, City University of Hong Kong, Kowloon, Hong Kong 999077, China; 3 Department of Civil Engineering, College of Engineering, Prince Sattam bin Abdulaziz University, Alkharj 16273, Saudi Arabia; a.bilal@psau.edu.sa; 4 Building & Construction Technology Department, Bayan University, Khartoum 210, Sudan; 5 Structural Engineering and Construction Management Department, Faculty of Engineering and Technology, Future University in Egypt, New Cairo 11745, Egypt; dina.mohamed@fue.edu.eg

**Keywords:** tungsten carbide, silica carbide, compressive strength, flexural strength, permeability

## Abstract

Flexural strength of concrete is an important property, especially for pavements. Concrete with higher flexural strength has fewer cracking and durability issues. Researchers use different materials, including fibers, polymers, and admixtures, to increase the flexural strength of concrete. Silicon carbide and tungsten carbide are some of the hardest materials on earth. In this research, the mechanical properties of carbide concrete composites were investigated. The silicon carbide and tungsten carbide at different percentages (1%, 2%, 3%, and 4%) by weight of cement along with hybrid silicon carbide and tungsten carbide (2% and 4%) were used to produce eleven mixes of concrete composites. The mechanical tests, including a compressive strength test and flexural strength test, along with the rapid chloride permeability test (RCPT), were conducted. It was concluded that mechanical properties were enhanced by increasing the percentages of both individual and hybrid carbides. The compressive strength was increased by 17% using 4% tungsten carbide, while flexural strength was increased by 39% at 4% tungsten carbide. The significant effect of carbides on flexural strength was also corroborated by ANOVA analysis. The improvement in flexural strength makes both carbides desirable for use in concrete pavement. Additionally, the permeability, the leading cause of durability issues, was reduced considerably by using tungsten carbide. It was concluded that both carbides provide promising results by enhancing the mechanical properties of concrete and are compatible with concrete to produce composites.

## 1. Introduction

Concrete is generally the most utilized construction material. It is a user-friendly, economical, easily moldable, and highly efficient structural material. However, it has some drawbacks, such as low modulus of elasticity, inconsiderable elastic range, low tensile and flexural strength, and permeability (due to which corrosion is susceptible). Therefore, concrete is usually reinforced to overcome such demerits using metals, polymers, and fibers [1,2]. There is a need to explore hard materials other than steel, such as various metallic carbides, in powder and coarse form to reinforce concrete properties.

Metallic carbides can exist naturally or are produced as byproducts of different industries. However, carbides have distinctive properties such as hardness, abrasiveness, tensile strength, neutron attenuation, heat resistance, and chemical inertness. They are added in metal to make the composites hard, corrosion and chemical resistant, and manufacture different tools [3]. Carbides are also being added in ceramics to produce ceramets to achieve better results [4]. The researchers initiated the investigation of these carbides as concrete admixtures. Adamu et al. showed that using calcium carbide residue along SCM can reduce cement consumption in concrete [5]. Kelechi et al. also confirmed the better durability of such carbides [6]. Researchers used different carbides in various forms, e.g., nanoparticles, powder, and flakes or fibers. The size and shape of carbides affect their properties in composites.

Silicon carbide (SiC) is incredibly hard and found scarcely in nature; however, it is a synthesized crystalline compound of silicon and carbon. It has high strength and chemical inertness. Silicon carbide produces strong materials such as sandpapers, crushing equipment, and cutting machinery [7].

When used in mortars and concretes, SiC decreases the fluidity and exhibits lower early strength but higher late strength. It reduces the shrinkage rate of concrete. The cement paste hydration process is affected, and the microstructure is densified by SiC addition [8]. The use of silicon carbide in the cement and concrete industry is a positive step toward sustainable development. SiC is also used in bulletproof vests [9], armors [10], and blast protection composites [11].

Silicon carbides are also found compatible to make aggregates for concrete. As sand replacement material, they improve the microstructure of concrete [12]. Pundienė et al. found that SiC increases density, compressive strength, fire resistance, and decreased concrete deformations [13]. Silicon carbide also increases sulfuric acid resistance. Kumar et al. proved that SiC increased polymer composites’ hardness and abrasion resistance [14].

Recently SiC was investigated for concrete production pertaining to particular applications [12,15,16]. SiC is a neutron attenuator and detector [17], and SiC is a radiation-tolerant ceramic. Woo et al. used SiC in concrete to increase thermal conductivity and freeze and thaw resistance [18]. Silicon Carbide in powder form significantly increases compressive and tensile strength [19].

Tungsten carbide (WC) is useful because it is a radiation protection material. WC in nano powder form provides higher protection from radiation and better compressive strength. Moreover, it has excellent wear resistance and hardness [20] and excellent chemical stability [21]. In addition, WC is a temperature-resistant and refractory material. It is extremely hard and has a stiffness of 18–22 GPa and Young’s modulus of 700 GPa [22,23].

The WC network causes specific plasticity and a high range of ductility properties while preserving the high stiffness. It has high corrosion resistance, and when exposed to the air, WC shows corrosion signs at temperatures over 600 °C. WC is also an electromagnetic radiation absorber [24]. 

WC is utilized alone or mixed with different metal composites to enhance strength. This ceramic carbide is also used to enhance the mechanical properties of different composites due to its strength, chemical stability, rigidity, and high-temperature resistance [25]. WC is a waste product of hard alloy metals [26], while SiC can also be found as a waste product [27].

Tungsten and tungsten carbides provide excellent gamma radiation shielding and neutron absorption [28]. WC provides a synergetic effect against wear and corrosion in composites and is used to increase the service life of the composites [29]. Fenghong et al. (2019) investigated that hybrid silicon carbide and tungsten carbide increase compressive strength, tensile strength, and wear resistance when added to an aluminum composite because both carbides are stiffer and stronger materials [30].

In this study, the effect of carbides (silicon carbide and tungsten carbide) on the mechanical properties and permeability of concrete composites were studied, and their compatibility with concrete was determined. Both carbides were added individually at 1%, 2%, 3%, and 4% of cement weight in concrete. The hybrid combination of both carbides at 2% and 4% was also used. The concrete density, compressive strength, corrosion properties, and flexural strength were investigated.

## 2. Experimentation and Methods

### 2.1. Materials

**Figure 1:**
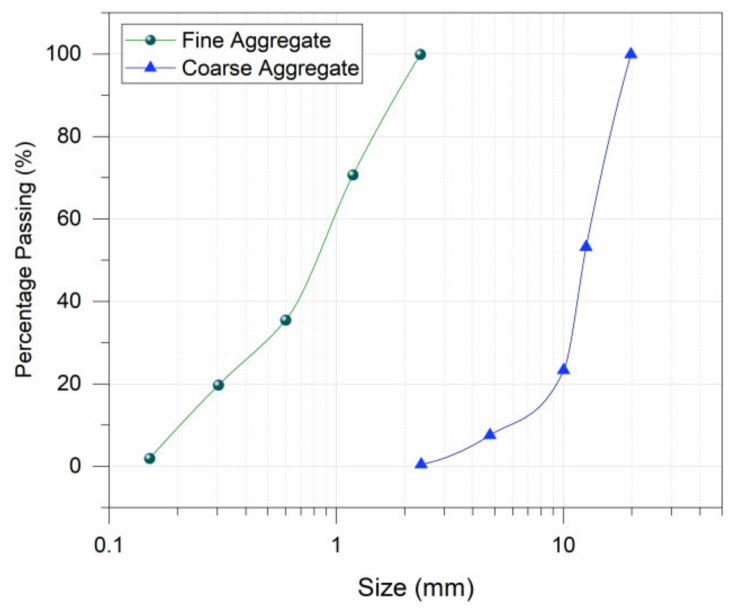
Gradation Curve for Fine aggregate (Sand) and Coarse Aggregate.

Ordinary Portland cement (Pioneer Cement) was used to prepare carbide concrete composites. Properties of cement used are presented in Table 1. Gradation curves of fine and coarse aggregate used are presented in Figure 1.

**Figure 2:**
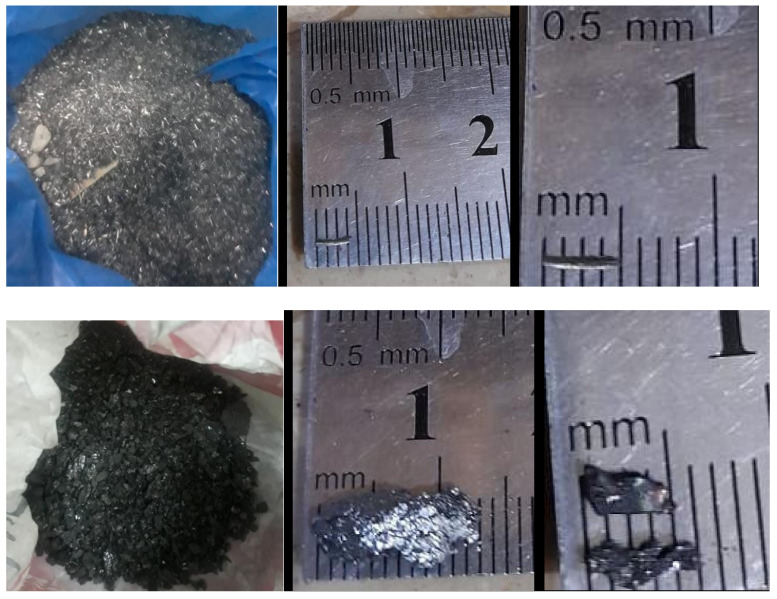
Tungsten carbide (**top**) and silicon carbide (**bottom**).

Figure 2 shows the carbides used in the study. WC, the small grey fiber in appearance, was almost small fiber size with an average diameter of 150 microns and 3 mm long. While SiC, the lustrous black flakes had lengths ranging from 3 to 13 mm and width 2 to 4 mm. WC was obtained from Deen Dye makers (Karachi, Pakistan), and SiC was obtained from F.S. Corporation (Lahore, Pakistan). Polycarboxylate-based superplasticizer was utilized to keep up high workability. 

The color and true density (density excluding pores) of silicon carbide and tungsten carbide used in the investigation are presented in Table 2. 

### 2.2. Sample Preparation and Testing

A total of 11 mixes of concrete were produced, as shown in Table 3. The concrete was produced by adding carbides (tungsten and silicon carbides) at different percentages of (1%, 2%, 3%, and 4%) by the weight of cement and hybrid carbide WC + SiC (1% + 1% and 2% + 2%). 

For each mix preparation, sand (fine aggregate) and crushed stone (coarse aggregate) were dry mixed for 1 min. Cement, carbides, and half of the water were added and mixed for 2 min. After a 1-min gap, the remaining half water mixed with superplasticizer was added and mixed for a further 2 min. A constant water–cement (w/c) and polycarboxylic-based superplasticizer content were used. 

Mixes were poured into molds and compacted by a vibrator. After twenty-four hours, samples were demolded and cured in water until the 28th day of casting.

#### 2.2.1. Fresh Density

The bulk density of each fresh concrete specimen was calculated by taking the ratio of the weight of four samples to the volume of samples from each batch. For this purpose, after casting of concrete, the pans with known volumes were filled, compacted, and weighed. Concrete mix density was determined within a short time after its production.

#### 2.2.2. Mechanical Testing

**Figure 3:**
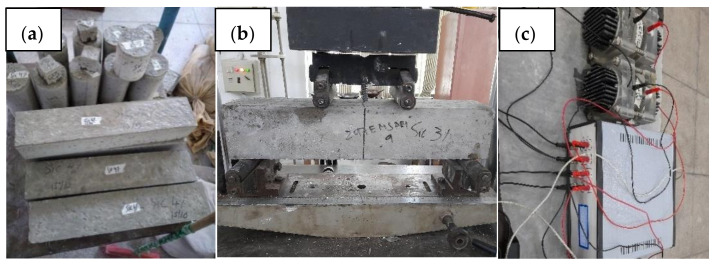
Experimental scheme (**a**) samples, (**b**) mechanical testing, (**c**) RCPT test setup.

Four cylinders (4 × 8 inches) were cast for each batch for the compressive strength test and four prisms (4 × 4 × 20 inches) for the flexural strength test. Figure 3 shows the samples and equipment (for mechanical and RCPT tests) used in experimentation. The mechanical tests were performed at 28 days of curing by applying four-point flexural test on prisms to find the flexural strength.

#### 2.2.3. RCPT Testing

Two cylinders (4 × 2 inches) were used to conduct a rapid chloride permeability test (RCPT). RCPT provides information about the permeability of the concrete to allow chloride ions to pass through it under 60-volt potential. For this purpose, two of 2 × 4 inches cylinders are first prepared using a vacuum desiccator and water. After sample preparation, the sample was fixed between two cells, one with NaOH solution and the other with NaCl solution. Voltage is applied to let the chloride ion pass through 2-inch concrete samples. The charge passed (coulombs) during 6 h indicates the permeability of the concrete. Finally, the rapid chloride permeability test (RCPT) was carried out on concrete samples. 

#### 2.2.4. Field Emission Electron Microscopy

FESEM images were studied to find out the reasons for the behavior of carbide composites. For this purpose, images at different magnifications at 10 KV EHT were obtained. The images found better for reasoning purpose was used in the study.

## 3. Results and Discussions

The results obtained through testing on concrete samples are presented and analyzed. 

### 3.1. Density

**Figure 4:**
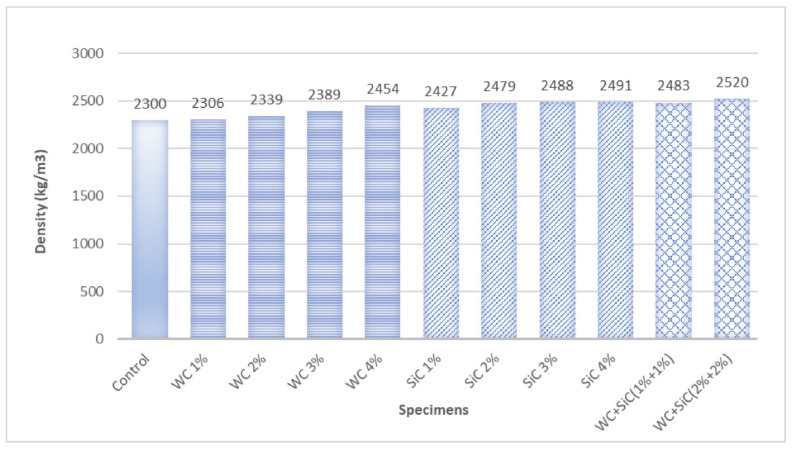
Density of all samples.

Figure 4 presents the density of all samples. The density of concrete increased with an increase in the carbides percentage, both WC and SiC, as shown in Figure 4. The maximum density was obtained at 4% of individual and hybrid carbides and hybrid carbides. Density was increased slightly by increasing the percentage of both carbides in the concrete. Because lower percentages of both carbides were used, the density was not significantly changed. However, the increase was more prominent due to SiC than for WC. The hybrid mix showed the highest density, and it might be due to particle packing. Jeon et al. also confirmed an increase in density by using SiC in concrete [31]. The typical density of SiC and especially WC are higher than concrete constituents. That might be the reason that carbides increased the density of concrete, even when used in low percentages.

### 3.2. Compressive Strength

**Figure 5:**
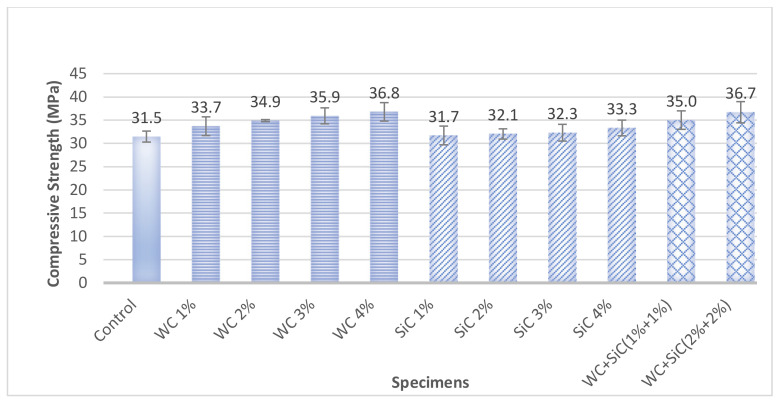
Compressive Strength of Specimens at 28 days.

Figure 5 presents the compressive strength of carbide concrete composites. The compressive strength of concrete increased slightly by increasing the percentage of both individual and hybrid carbides up to 4%. An increase in compressive strength up to 17% at 4% of WC was noticed. The increase in compressive strength can be attributed to the small fiber-like structure of the WC used in the investigation. It can be inferred that WC small fiber can be compared to steel fibers for effectiveness and cost. Usually, researchers use the fiber at 1–3% of concrete by volume [1]. However, the carbides used in this investigation were additions with respect to the weight of cement; hence, they may be much lower in quantity than ordinary fibers. This approach was devised because using materials at a certain cement percentage is a more handy and easy job.

SiC is also one of the hardest ceramic materials [7,32]. It was used in the form of lustrous back flakes. The increase in compressive strength was up to 6% at 4% of SiC. Strength increase may be attributed to their higher abrasive nature [14], leading to high resistance to motion, resulting in a significant increase in strength. Ren et al. and Jeon et al. also corroborated an increase in compressive strength using SiC [27,31]. 

It was observed that the hybrid WC and SiC in concrete enhanced the compressive strength considerably. It might be due to better interphase developed due to carbides and higher density due to better packing of particles, leaving fewer voids behind. 

The enhancement in compressive strength may also be attributed to the carbides’ intrinsic compressive strengths as concrete constituents.

### 3.3. Flexural Strength

Strength is the most important property of concrete. The concrete generally has weak flexural strength [33,34,35]. Flexural strength plays a very important role in the design of concrete pavements. The increase in the tensile/flexural strength is a desirable goal for the researchers. Concretes with higher tensile/flexural strengths are less prone to cracks and durability issues. Additionally, flexural strength is the most important property for concrete pavement design. Researchers nowadays are trying to improve flexural strength by using different admixtures, fibers, and other techniques for pavement concrete [36,37,38].

**Figure 6:**
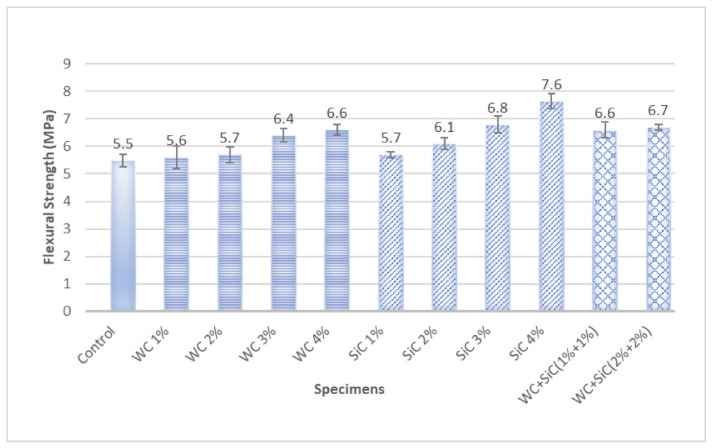
Flexural Strength of Specimens at 28 days.

The impact of carbides in concrete with different percentages on flexural quality is shown in Figure 6.

The flexural strength of concrete was significantly increased by increasing both individual and hybrid carbides. However, SiC showed a very high flexural strength than WC, unlike compressive strength. WC showed an increase in flexural strength up to 20%, while SiC showed a 39% increase in strength. It can be easily attributed to the shapes, size, and form used during testing. WC has very high tensile strength, but it was used in small fiber form. It increased the flexural strength considerably, even when used in a minimal amount. Flakes of SiC flakes did not allow prism to fail easily, and they provided enough reinforcement for increasing the flexural strength of concrete. Although it has very high tensile/flexural strength, WC could not transfer tensile stresses effectively due to its tiny size of 3 to 4 mm. The higher intrinsic strength of carbides led to the higher flexural strength of composites.

Additionally, the abrasive resistance did not allow composite particles to move away easily [14]. It resulted in a higher strength of composites. However, unlike SiC, the effect was not significant for WC due to WC’s minimal length and much lower volume as compared to SiC at the same mass. It is because the density of WC is almost 4.5 times higher than SiC.

The hybrid carbide WC and SiC showed high flexural strength. WC is usually mixed with other metals because of its capacity for synergistic effects [39,40,41,42]. Lin et al. found a synergistic effect of WC and SiC on a composite [40]. They found a synergistic effect of both carbides on the strengthening of composite. The better results of WC + SiC composites might be attributed to the better interphase and synergetic effect of carbide composite constituents.

**Figure 7:**
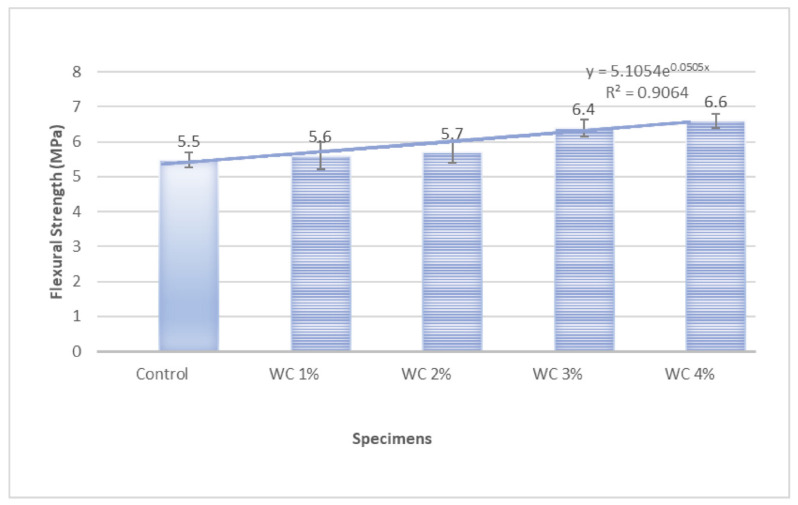
Flexural Strength Variation with the increase in WC percentage.

**Figure 8:**
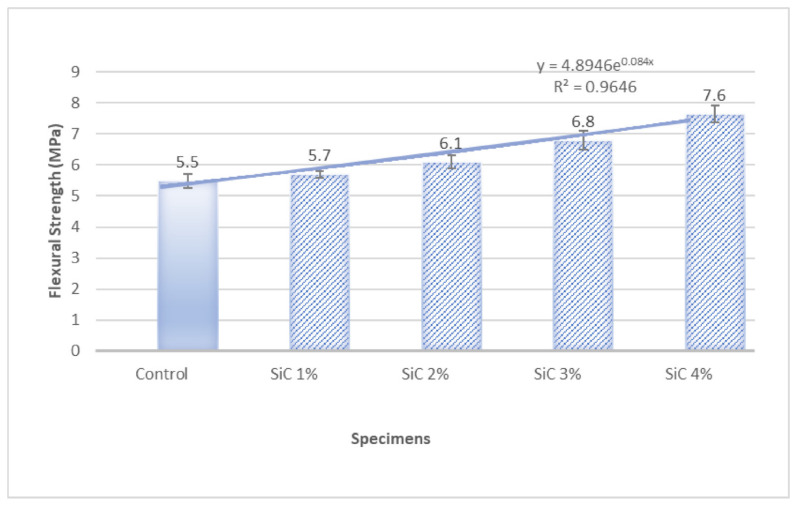
Flexural Strength Variation with the increase in SiC percentage.

ANOVA analysis conducted to find the role of fiber percentage in improving flexural strength also corroborated the significant effect of both carbides percentage on flexural strength. It can be said by 95% confidence (error chance < 5%) that by changing the percentage of carbides, flexural strength was changed significantly. *p* < 0.05 (and near to 0) indicates that the alternate hypothesis is true, and the effect of fiber percentage is so significant that it can not be just by chance. For WC, the *p*-value is 0.028 (Figure 7 and Table 4), and for SiC, the *p*-value is 0.010 (Figure 8 and Table 5). Therefore, both carbides have significant effects on flexural strengths.

Root mean square error (R^2^) value near 1 suggests a good fit of predicted and experimental values. Figure 7 shows that R^2^ is 0.906 for WC-containing composites, proving the good fit of the model developed. Figure 8 shows that R^2^ is 0.96 for SiC containing composites, proving the excellent fit of the model developed.

Barraza et al. found higher mechanical properties when SiC micro flakes were used in geopolymer concrete [43]. Improved mechanical properties were obtained because of micro flakes rather than geopolymer interphase. SiC flake improves flexural strength.

### 3.4. Rapid Chloride Permeability Test

The permeability of concrete is a critical property that controls other durability issues [44]. Higher permeability relates to lower durability, high corrosion potential, and other durability issues. The RCPT test is the standard test method for indicating the chloride permeability and potential to corrosion of steel. After achieving the best results for 4% of individual and hybrid carbides, these concrete carbides were tested for rapid chloride permeability. 

**Figure 9:**
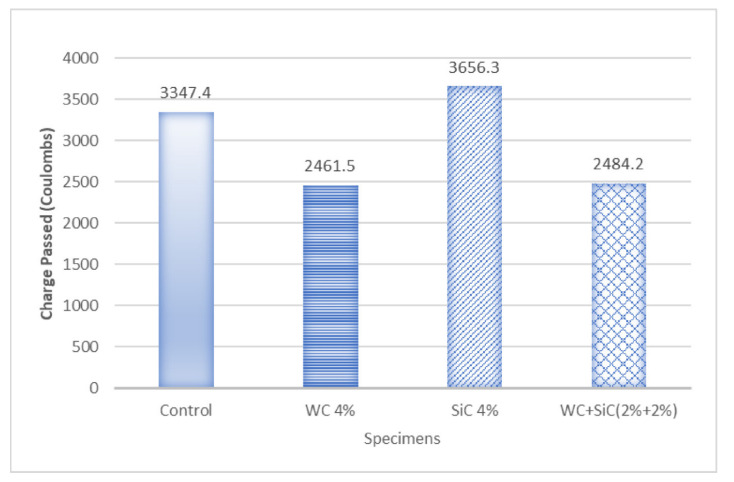
Rapid Chloride Permeability Test of Specimens.

Figure 9 depicts RCPT results by comparing average charges passed through carbides concrete composites and plain concrete. This test concluded that the maximum charge was passed through SiC 4%, and the minimum charge was passed through WC 4%.

The results obtained showed that WC showed maximum resistance against chloride permeability. WC reduced the permeability of concrete composite when used alone or in the hybrid mix. WC is very dense and does not allow ions to pass through it. Therefore, even in small amounts, WC provides barriers to ions passing through concrete.

However, silicon carbide showed higher permeability. SiC flakes increased the permeability of the composite due to its porosity [27]. The porosity of SiC can explain this unexpected result of SiC, which was used in the form of flakes. Hybrid carbide composite abated the permeability because of their synergistic effect and better density.

### 3.5. Field Emission Scanning Electron Microscopy

**Figure 10:**
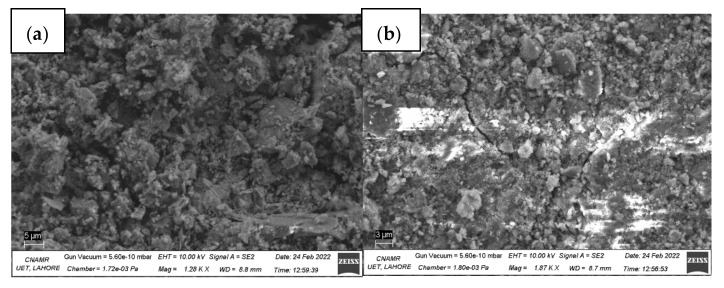

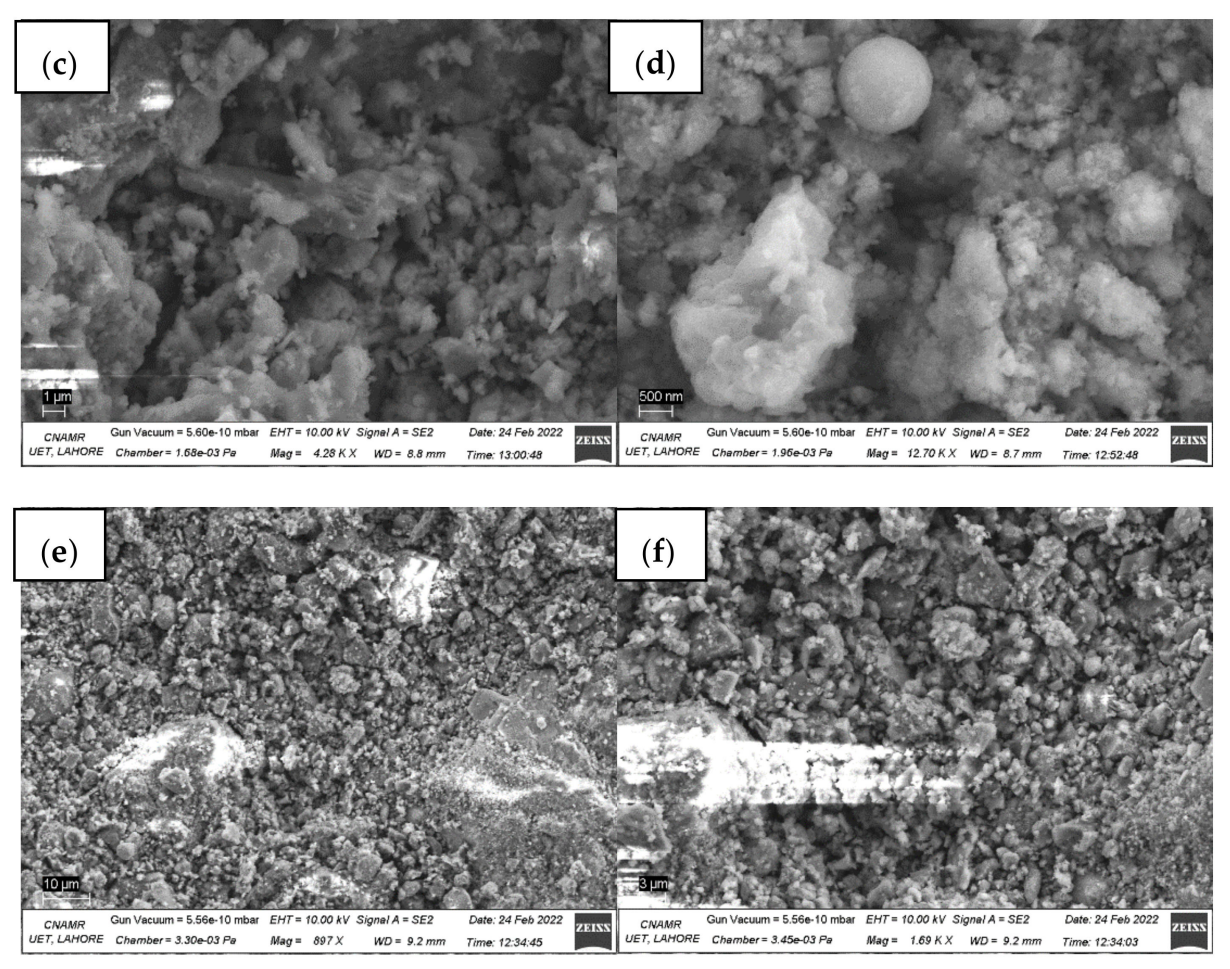
FESEM results for (**a**) control sample, (**b**–**d**) WC4% sample and (**e**,**f**) SiC4% samples.

Figure 10 shows the Field emission scanning electron microscope images for control (a), WC4% (b–d), and SiC4% (e,f) samples.

In Figure 10b, the needle-like metallic luster under the hydration products shows the presence of WC whiskers. It is also evident that this needle passes under the very fine crack, thus bridging the crack. WC does not show obvious ITZ, which may be the reason for better strengths caused by WC addition. WC whisker looks similar to solid metal, and no porosity is apparent. It also seems that WC attracts hydration products. In Figure 10d, a circle of white salt-like hydration products is visible that may have formed at the end of the WC needle. Good interbond and no clear ITZ show better WC compatibility with concrete, resulting in better strengths. No noticeable porosity results in low permeability.

SiC is more lustrous and has a rough surface with some noticeable pores in Figure 10e and especially in Figure 10f. SiC flake has an irregular surface and shape (Figure 10f). The porosity in SiC flake (evident in the figure) contributes to a higher samples’ permeability. The roughness of the surface along porosity determines a better bond and may cause higher flexural strength.

## 4. Conclusions

In the present study, the influence of two of the hardest materials on earth, silicon carbide (SiC) and tungsten carbide (WC), on the compressive strength, flexural strength, and permeability of concrete was investigated. Both carbides were used individually and in the hybrid form added up to 4% of cement weight to produce carbide concrete composites.

The increase in the percentage of both carbides, individually and hybrid form, increased the compressive strength and flexural strength of concrete. Though both carbides increased the compressive strength, WC concrete composites showed higher compressive strength (i.e., 17%) than SiC composites (6% increase in compressive strength). Contrarily, SiC showed very high flexural strength (39%) compared to WC (20%), although both carbides enhanced flexural strength noticeably. The ANOVA analysis proved the significant effect of both carbide contents on the flexural strength of concrete. This property makes WC and especially SiC desirable for pavement concrete.

Additionally, the Rapid chloride permeability of flaky SiC concrete composite was higher. However, WC and hybrid composites showed relatively lower permeability when used at 4%. FESEM images also verify the results. It is evident that both tungsten and carbides enhance the properties and are quite compatible with concrete. However, hybrid carbide composites provided a synergetic effect by enhancing the mechanical strength, especially flexural strength, and mitigating the permeability. 

## 5. Future Recommendations

The composite properties were enhanced directly by increasing percentages of carbides up to 4% addition. It implies that investigation can be carried out on higher percentages of both carbides. The work can be further extended to concrete composite for other unique properties of their constituent carbides, including neutron shielding, bulletproofing, electromagnetic radiation barriers used in nuclear reactors, and 3D printing. The positive results, if obtained, may pave a path for the production of special performance-based cement and concrete. 

## Figures and Tables

**Table 1:** Chemical composition and properties of cement.

Sr.	Composition/Properties	Result
1	% SiO_2_	19.67
2	% Al_2_O_3_	4.90
3	% Fe_2_O_3_	3.46
4	% CaO	63.11
5	% MgO	1.85
6	% K_2_O	0.96
7	% Na_2_O	0.16
8	% SO_3_	2.71
9	% Cl	0.012
10	Blain, cm^2^/gm	3078
11	Consistency	30
12	Initial Setting Time	110 min
13	Final Setting Time	220 min
14	Specific Gravity	3.13

**Table 2:** Properties of WC and SiC.

Elements	Color	True Density
SiC	Greyish-black lustrous solid	3216 kg/m^3^
WC	Grey	15,630 kg/m^3^

**Table 3:** Composition of Prepared Samples.

	Mix ID	Cement (kg/m^3^)	Sand (kg/m^3^)	Coarse Aggregate (kg/m^3^)	Water (kg/m^3^)	SP (kg/m^3^)	WC (kg/m^3^)	SiC (kg/m^3^)
1	Control	430	688	1118	190	4.3	-	-
2	WC1%	430	688	1118	190	4.3	4.3	-
3	WC2%	430	688	1118	190	4.3	8.6	-
4	WC3%	430	688	1118	190	4.3	12.3	-
5	WC4%	430	688	1118	190	4.3	17.2	-
6	SiC 1%	430	688	1118	190	4.3	-	4.3
7	SiC 2%	430	688	1118	190	4.3	-	8.6
8	SiC 3%	430	688	1118	190	4.3	-	12.3
9	SiC 4%	430	688	1118	190	4.3	-	17.2
10	WC + SiC(1% + 1%)	430	688	1118	190	4.3	4.3	4.3
11	WC + SiC(2% + 2%)	430	688	1118	190	4.3	8.6	8.6

**Table 4:** ANOVA for Flexural Strength Variation with increased WC percentage.

Summary							
**Groups**	**Count**	**Sum**	**Average**	**Variance**			
Control	4	21.936	5.484	0.05012376			
SiC1%	4	23.1117	5.777925	0.00687578			
SiC2%	4	24.6323	6.158075	0.43214339			
SiC2%	4	24.6323	6.158075	0.43214339			
SiC3%	4	25.0893	6.272325	0.30265701			
SiC4%	4	30.5733	7.643325	2.04863465			
**ANOVA**							
**Source of Variation**	**SS**	**df**	**MS**	**F**	***p*** **-value**	**F crit**	**Effect**
Between Groups	11.03379	4	2.758447	4.855677	0.0103038	3.055568	Significant
Within Groups	8.521304	15	0.568087				
Total	19.55509	19					

**Table 5:** ANOVA for Flexural Strength Variation with the increase in SiC percentage.

Summary							
**Groups**	**Count**	**Sum**	**Average**	**Variance**			
Control	4	21.936	5.484	0.05012376			
WC1%	4	22.4844	5.6211	0.22555692			
WC2%	4	22.1766	5.54415	0.08482964			
WC3%	4	25.592	6.398	1.09019178			
WC4%	4	26.4146	6.60365	0.06892017			
**ANOVA**							
**Source of Variation**	**SS**	**df**	**MS**	**F**	***p*** **-value**	**F crit**	**Effect**
Between Groups	4.464174	4	1.11604359	3.67210858	0.0281423	3.055568	Significant
Within Groups	4.558867	15	0.30392445				
Total	9.023041	19					

## Data Availability

The data presented in this study are available upon request from the corresponding author.

## Nomenclature

Silicon carbide	(SiC)
Tungsten carbide	(WC)
Rapid chloride permeability test	(RCPT)
